# The Challenges of Scientific Publishing: An Editor‐In‐Chief's Perspective

**DOI:** 10.1111/fcp.70105

**Published:** 2026-07-05

**Authors:** Luc Zimmer, Pascal Bousquet

**Affiliations:** ^1^ Centre de Recherche en Neurosciences de Lyon Université Lyon 1, Inserm, CNRS Lyon France; ^2^ Hospices Civils de Lyon Lyon France; ^3^ Institut National des Sciences et Techniques Nucléaires (INSTN) Saclay France; ^4^ Faculté de Médecine Université de Strasbourg Strasbourg France

**Keywords:** bibliometrics, paper mill, publication ethics, retraction, scientific misconduct, scientific publications

## Abstract

The current and former editors‐in‐chief of the Fundamental & Clinical Pharmacology journal share their experience and perspectives regarding the present landscape of scientific publishing and the challenges and threats it faces. Several challenges are identified, three of which are of particular significance. The first is how to manage the growing number of submissions. This is a global trend: The number of indexed articles has increased by 50% in less than 10 years, and the challenge of finding efficient and motivated reviewers is particularly acute. The second challenge is how the impact factor has come to be the sole quality criterion for a scientific journal, and by extension for evaluating researchers: Might there not be alternatives? The third challenge is publication fraud, where the editor‐in‐chief is invariably exposed to endless attempts at manipulation. However, there are fortunately now tools to help combat this. Of course, the editor‐in‐chief's ultimate weapon is the power to retract a suspicious article, but this is not so simple and is not without consequences.

## 1 Introduction

Scientific publishing is undergoing deep changes, which some perceive as a crisis and others as an opportunity. Publication chiefly comprises articles in standardized formats in specialized journals. An article first undergoes review by a panel of experts, who may reject it or request revision of form or content before acceptance. The scientific article has long been the definitive medium for the transmission and exchange of validated data. However, changes in recent years incur challenges for editorial boards. The primary challenge is the increase in the number of articles submitted worldwide to an ever‐expanding array of journals. The problem is how to assess them all, meticulously and impartially. The second challenge is to move away from the current impact‐factor model for journal ranking, which is imperfect and inappropriately quantitative. The final challenge is to combat scientific fraud, which jeopardizes the credibility of scientific publishing. These challenges are addressed here from the perspective of a current and former editor‐in‐chief of Fundamental & Clinical Pharmacology (FCP).

## 2 Challenge #1: Managing the Growing Number of Scientific Articles

### 2.1 Global Increase in the Number of Submissions

Publication is the culmination of research. It is governed by ethical codes established in Robert K. Merton's seminal article of 1942 [1]. The fundamental principle is “organized skepticism,” relying on peer review, with each article meticulously evaluated to ensure the highest standards of rigor and integrity. Scientific publishing is experiencing a series of revolutionary developments. First, the number of articles is increasing. More than 2.8 million articles are indexed each year in the main databases, Web of Science and Scopus, representing a 50% increase between 2016 and 2024 [2]. The Dimensions publishing platform lists 20 000 scientific publications per day, or 6 million per year, taking all languages and disciplines together [3].

This trend is confirmed in FCP. In 2024, 500 articles were submitted, with a third in the clinical pharmacology section and the rest in fundamental and experimental pharmacology. The total number was relatively stable, at approximately 120 per year for 2010–2012, but increased significantly from 2020, exceeding 500 articles per year from 2022 onward. Examination of the geographical origins is illuminating. Several countries have adopted very proactive investment policies, in terms of both the number of researchers and the creation of cutting‐edge public and private research centers. China and India are at the forefront, the annual number of articles published by the former now exceeding that of the United States [4]. This is indicative of an increased interest in biomedical sciences, which are a driving force for progress in health and a source of well‐being and wealth. However, certain countries experiencing rapid scientific growth prioritize quantity over quality [5].

Concurrently, prominent scientific publishers such as Elsevier, Springer Nature, Wiley‐Blackwell, and Taylor & Francis have increased their number of journals tenfold in two decades [6], necessitating an escalating number of reviewers. Another issue concerns “special issues”: For example, MDPI boasts a multitude of journals that together publish hundreds of special issues every year [7]. Editorial boards have to manage this flood of submissions—and conventional practices are proving inadequate, as seen below.

### 2.2 Finding Reviewers Is Increasingly Difficult

The number of articles to be processed, competently and equitably, is a major challenge, given the difficulty of ensuring effective, reliable, rigorous, and prompt peer review. In most journals, including FCP, peer review is a voluntary service to the scientific community. Researchers often undertake additional tasks, not always recognized as part of their work [8]. The approach is exceedingly time‐consuming and cannot be extended in its current form. There are also scientific challenges: It can be very difficult to find competent independent experts with no conflicts of interest and willing to give their time. Journals frequently contact 30 or 40 potential reviewers to secure agreement from just 2 (the minimum number for satisfactory peer review). In 2024, FCP approached more than 2000 experts as potential reviewers, who rejected 87% of submissions and accepted the other 13% following revision. These figures are comparable with those reported by many other journal editors [9]. The worry is that the peer review system may break down under the volume of scientific output and the number of manuscripts. The primary consequence is an ever‐longer review process before publication, whereas journals also highlight the promptness of their initial response (rejection or review), which now takes only a few days in most cases.

#### 2.2.1 Retaining Reviewers

Some editorial teams endeavor to maintain the services of reviewers by a variety of tactics. First, reviewers can add their reviewing activity to their author profile on Web of Science. For example, journals can export a researcher's review data to the “Peer review” section via Crossref [10]. Some platforms offer their services to journals, either for a fee or free of charge, to enable reviewers to access their services. Some journals attribute evaluation certificates to reviewers. The reviewer's ORCID identifier allows the journal to document their reviews on their ORCID or WoS Reviewer Recognition Service profiles [11]. Others acknowledge reviewers at the end of the year, publishing their names. Exceptionally, the list of acknowledgements is published with a Digital Object Identifier (DOI), enhancing visibility in article databases. Other journals publish the names of reviewers on the publication page, or even publish a signed review alongside the article, including the authors' responses. This approach has the merit of making the work of reviewers, which usually goes unrecognized, visible, and more valuable; Frontiers, eLife Sciences, and PeerJ have adopted this type of recognition.

Payment for reviewing is more controversial. Scientific publishing is highly lucrative, and it might not be unreasonable for reviewers to be paid for their time and effort. Remunerating researchers and experts on an ad hoc basis is already commonplace for project evaluation by academic or private funders. Few journals, however, offer any direct or indirect financial compensation for their review process [12]. Some occasionally offer discounts on publication fees, or even publication credits; direct remuneration is much less common. Two journals, Critical Care Medicine and Biology Open, experimented with payments up to $250 when reviews are completed promptly without compromising quality. The conclusions of these two studies were not identical, but generally, paid reviews were completed more quickly than unpaid reviews [13], without a difference in quality. The question therefore is whether there is any added value in paying for reviews. The other issue relates to reviewers' professional interests: If they were not involved in reviewing, they would be disconnected from cutting‐edge research, which is essential professionally. It is reasonable to entertain a degree of skepticism regarding this matter.

#### 2.2.2 Replace Reviewers by Artificial Intelligence (AI)?

Until recently, the idea that AI could replace human reviewers seemed outlandish [14]. However, rapid progress has made it conceivable. Preliminary studies compared the evaluation of clinical cases by a human expert and by AI, with impressive results [15]. The editor‐in‐chief of NEJM AI is convinced that AI will soon become unavoidable to compensate for the problems of recruiting high‐caliber reviewers [16]. He even announced that his journal will be able to make decisions within 7 days. To achieve this, the editorial board will consider three opinions: of associate editor, of ChatGPT5, and of Gemini 2.5. At this stage, a human expert is still comparing their own opinion with AI. Substandard AI is likely already being used by low‐quality predatory journals. Several companies are developing tools for publishers in conjunction with AI‐powered reviewing tools [17]. The question is no longer whether, but when AI will replace reviewers. This is obviously a cause for concern, jeopardizing collegiality and scientific trust, among the pillars of scientific ethics according to Merton in 1942 [1]. It is a fact that AI systems can detect plagiarism, identify statistical inconsistencies, and check manuscripts for formal compliance. This helps to reduce the workload of human reviewers. However, these benefits are not without limitations: Generative models are prone to “hallucinations,” producing assessments that are plausible but factually incorrect, including references to nonexistent sources or unfounded methodological criticisms. Furthermore, there is a lack of algorithmic transparency, meaning the exact criteria that lead to critical judgments are often opaque, making it difficult to conduct a scientific audit of decisions. Additionally, these tools tend to reproduce, or even amplify, existing biases within the publication system, such as the overvaluation of highly cited articles or dominant trends, which can be detrimental to negative results or less‐cited works. It is important to understand that AI systems are designed to process data based on what they have already learned. This means they may not be able to identify innovations, scientific advancements, or breakthroughs. So, what does “progress” mean for AI? AI is a powerful tool that selects a large amount of data and combined ideas based on the opinion of experts. So, there is a chance that AI might suggest ways that help scientists make real progress. However, reviewing a publication in depth is a cognitive process that currently eludes AI, at least in its current state. In this context, it should be viewed as a tool to assist human editorial judgment and not as an autonomous arbiter.

## 3 Challenge #2: Replacing the Impact Factor (IF)

### 3.1 The Limitations of the IF

The academic research community has always sought to standardize publication metrics for evaluating researchers and institutions [18]. Several metrics can be used, and notably the IF, defined by its creators in 1975 [19] as “[the] average number of times documents published in a journal in the past two years have been cited in the current year”: i.e., IF for year *x* = [number of citations in year *x*]/[number of publications in year *x* − 1 + number of publications in year *x* − 2]. It was originally conceived as a metric of journals' impact. It shows significant variation, according to the journal's field, nature, and degree of specialization. Consequently, its validity has always been disputed [20]. Nevertheless, it came to be employed for a multitude of other purposes, including as a proxy for the reputation of researchers, laboratories, and research institutions. There seems to be a consensus that the number of articles a researcher publishes in high‐IF journals is indicative of their expertise. However, the IF represents simply the number of citations made to articles, which is not the same thing as intrinsic value in terms of methodological quality, scientific relevance, or originality. Moreover, IFs vary significantly between research fields. The field of pharmacology, along with the pharmaceutical sciences to which FCP belongs, “underperforms” in citation rates compared to other disciplines. Comparison of IFs across different academic disciplines is meaningless. A journal's IF can also be boosted by the “inflationary” effect of a few much‐cited articles, while others may be cited very little. Finally, IF is calculated from just the two most recent years, which fails to consider original innovative articles that are cited only much later, when an initially niche topic has become of interest to a broader community of readers.

### 3.2 Beyond the IF?

For many years, the scientific community has talked about replacing or at the very least supplementing the IF with other indices and metrics. The San Francisco Declaration (also known as DORA, or Declaration on Research Assessment) in 2012 called for the IF not to be used as the primary measure of individual researcher quality [21]. After this, the Leiden Manifesto and the Hong Kong Principles were published in 2015 and 2020, respectively [22, 23]. By 2025, the number of signatories to the DORA initiative had reached nearly 22 300, encompassing organizations and individuals from 159 countries worldwide. Certain universities have taken the initiative. The University of Ghent in Belgium [24] and Utrecht University in the Netherlands [25] have ceased using the IF as an evaluation metric for individuals, and the Swiss National Science Foundation asks researchers in biology and medicine to include only their four most significant contributions to science in their CVs, irrespective of the IF of the journals in which they were published [26].

But are there alternatives? The h5‐index (Google Scholar Metrics) or CiteScore (Scopus) evaluates journals over a period of 5 or 3 years rather than 2 years. Scimago Journal Rank is based on the number of citations but assigns greater weight to those in journals with a greater reputation. The Eigenfactor Score (Web of Science) weights citations according to the influence of the journal in which they are made, except for self‐citations. The objective of these alternatives is threefold: to weight citation quality, to broaden the time window for measuring citations, and, for Altimetrics, to integrate the media impact of article citations [27]. Awaiting the phase‐out of IF, editors‐in‐chief track Altimetrics without overrating it and no longer communicate purely to attract submissions.

## 4 Challenge #3: Combating Publication Fraud

### 4.1 Scientific Misconduct, a Life‐Threatening Menace for a Scientific Journal

What is the difference between misconduct and error? Scientific misconduct has been defined as “falsification, fabrication, or plagiarism committed in the context of a research proposal, the conduct of research, the evaluation of research, or the reporting of research results” [28]. Conversely, error is inherent to the scientific method, potentially giving rise to subsequent correction without impugning the integrity of the authors, whereas fraud erodes the fundamental trust between researchers, impeding the dissemination of knowledge itself.

The editor‐in‐chief of a biomedical scientific journal such as FCP is at the forefront of efforts to combat a wide range of misconduct. This is now a primary concern when receiving any submission. The phenomenon encompasses fabrication of experimental data and manipulation of statistical tests, as well as plagiarism of data or text without proper citation of the original publication. Redundant publication consists in multiple publications of the same data in overlapping articles and even self‐plagiarism. Another frequent form of misconduct is the simultaneous submission of the same article to several journals. These fraudulent activities may be sporadically perpetrated by unethical researchers but can also be orchestrated on an industrial scale.

### 4.2 The Threat Posed to Integrity by Paper Mills

Paper mills are predatory, mercenary companies mass‐producing fraudulent articles. They sell their “scientific articles” to dishonest researchers seeking to augment their publication count in pursuit of career advancement [29]. Paying to secure a place as a co‐author is unfortunately common in academic publishing. The most prestigious and consequently the costliest places as a co‐author are first or last author. The cost of publication is increased further when an article has already been accepted by a journal. The articles, in general of poor quality, used to be formatted by PhDs employed by paper mills [30] but are now also simply written by AI. Unexpectedly, the growth of open data and open science practices, although essential for transparency and reproducibility, introduces new vulnerabilities that are exploited by paper mills. The widespread availability of datasets, protocols, and metadata therefore also facilitates large‐scale misuse. Paper mills now exploit these open resources to fabricate articles from scratch that include synthetic or manipulated data, which is difficult to detect within a saturated editorial workflow. The emergence of AI capable of generating “realistic” datasets exacerbates this risk: Trained on open repositories, it can produce results that meet expected statistical standards, but which do not correspond to experimental reality. This situation undermines a central pillar of open science: trust in shared data. It necessitates a rethink of data certification, traceability, and verification mechanisms, particularly in a context where fraud is becoming increasingly sophisticated.

The advent of these companies was precipitated in a burgeoning scientific milieu by the prevailing imperative to publish or perish, which is particularly pronounced in certain countries. Articles from paper mills now account for approximately 2% of all published articles: i.e., some 60 000 per year [31]. Paper mills operate clandestinely, but some companies are more transparent and attempt to directly bribe journal editors. For instance, one of the authors of this article has been approached on multiple occasions by companies based in Asia, offering substantial sums in exchange for a series of publications. Furthermore, some companies endeavor to get their own associate editors or reviewers appointed to editorial boards.

The latter approach illustrates an even more sophisticated form of predation that has recently emerged. “Review mills” are devoted to the production of low‐quality evaluations, typically accompanied by a request that the author cite specific articles, undoubtedly those of the researchers who paid for the service in question [32]. The objective is to enhance their citation metrics and thus their scientific esteem. Additionally, “citation mills” have emerged, with the objective of enhancing the IF of specific researchers by selling citations and introducing them into the publication circuit via paper mills or review mills [33].

Another issue is the possibility of relationships that violate scientific integrity between authors, reviewers, and, in some cases, associate editors, particularly in “special issues,” which are a format offered by many journals [34]. Clarivate found that a significant number of fraudulent articles and articles from paper mills were being published in special issues, the editor‐in‐chief of which was unaware of or else complicit in this misconduct [35]. Furthermore, it is customary for a journal to request that an author provide a shortlist of potential reviewers when submitting an article, and it is tempting to propose acquaintances likely to be favorable to the submission. Even more astonishing, some authors go so far as to create fake reviewer profiles, thereby ensuring the evaluation of their own article [36]. Editors‐in‐chief are now monitoring these practices and have found ways to counter them. Finally, certain types of articles lend themselves to extensive use of AI, “Letters to the Editor” being a prime example, and several scientific journals have recently suspended acceptance of these. This follows a notable upsurge in such “correspondence,” probably composed using AI tools with the selection of appropriate prompts [37].

### 4.3 Measures in Response to Paper Mills

The proliferation of articles from paper mills has prompted research institutions, publishers, and researchers to gather to find counterfires [38]. Meanwhile, major publishers have established their own scientific integrity departments, and they coordinate through the International Society for Technical and Medical Publishers' Integrity Hub [39]. Wiley, which publishes FCP, employs nearly 30 individuals in this department. Wiley, Elsevier, Frontiers, and Springer Nature are now equipped with software to screen submissions. The interfaces are based on increasingly sophisticated automated AI technologies that detect suspicious signs [40], developed either in‐house by large publishers or by start‐ups, the number of which is growing rapidly: Cactus, Clear Skies, and so forth. The precise mechanisms by which these tools function to check the integrity of publications are kept secret, as paper mills are increasingly ingenious in circumventing the most well‐known methods. New features are continually being incorporated to counteract the evolving tactics of fraudsters. The most prominent methods involve plagiarism and image manipulation detectors that indicate that the text has been written by traditional AI systems, such as ChatGPT or Google Gemini. Others can detect plagiarized text reworded by automated paraphrasing tools available online, such as SpinBot. Furthermore, author credibility is subject to rigorous scrutiny: publication history, retractions, consistency of affiliations, and authenticity of academic email domains.

### 4.4 Editorial Eyes Are Irreplaceable

Despite technological assistance, human judgment remains indispensable. The editor‐in‐chief cannot rely exclusively on anti‐fraud software: It can alert that an article presents a “high risk of originating from a paper mill,” but detection systems are not transparent, and the editor has little information to qualify or weigh up the alert. The ultimate decision regarding rejection, correction, or withdrawal lies with the editorial board. Rules of thumb are applied in the light of the experience and insights of other editors [41]. Drawing upon our own experience in this field, we found that 10%–15% of articles submitted to FCP were directly rejected for “ethical concerns” prior to any other form of evaluation. This finding is consistent with reports by other editors.

Detecting multiple submissions is difficult but crucial. It is estimated that 50% of duplicate submissions originate from paper mills, which, lacking any moral compass, seek simply to maximize the chances of success for their clients [42]. This is easily detectable when the journals belong to the same publisher, as their shared database provides that information. It is more difficult when submissions are made to several publishers; it is often a reviewer who has been asked to assess the same article for another journal who sounds the alert.

#### 4.4.1 Retraction: The Ultimate Weapon?

It is preferable to reject an article as early as possible, at submission. However, should the article have slipped past peer review and been validated by associate editors, there remains the nuclear option of retraction. Suspicious articles are frequently reported to the editor‐in‐chief by a whistleblower. Scientific communities are committed to identifying problematic articles by various means, including reports from community members or by detecting “weird” sentences that suggest the use of AI. The community has developed a suite of digital tools, including the Problematic Paper Screener [43]. But the process does not stop at identifying misconduct. Requests by scientists for corrections or even retractions often take time and are not always successful [44]. In a 2023 study analyzing more than 17 000 publications reported on PubPeer, only 21.5% were corrected [45]. However, scientific publishers are taking action: The number of retracted articles has never been higher than in recent years, with more than 10 000 retractions in 2023, double the figure for 2020 [46]. No publisher has been spared, not even the most well‐known. The alarm bells started ringing in 2023 when Hindawi, a branch of the publishing giant Wiley, retracted more than 8000 scientific articles [47]. Another shockwave occurred in April 2025 when nearly 700 articles were retracted in a single journal published by Sage [48]. When a single journal has hundreds of articles retracted, its reputation is inevitably tarnished. Worse still, its metrics can be fundamentally disputed. Clarivate, the company that owns Journal Citation Reports (JCR), removed 82 and 128 journals from the Web of Science database in 2023 and 2025, respectively. As a result, these journals found themselves without any IF, and a few of them were closed a few months later, as they were no longer attracting enough authors. Some observers are concerned that journals, subject to such “double jeopardy,” will be reluctant to pursue active policies of retracting problematic articles. This is not good tactics, because in fact, according to Clarivate, only one journal in a hundred would see its IF decrease, and very slightly at that—by less than 3% for half of the journals concerned [49]. The other difficulty is making corrections or retractions visible to future authors. In theory, the publisher sends the information to databases such as Web of Science or Scopus, but the update is not always immediate. This is a problem when readers use these databases and are not informed that an article has been retracted. A recent study in Learned Publishing [50] found that more than 920 000 scientific articles currently cite “problematic” articles, not taking retraction into account; furthermore, ChatGPT cites retracted articles as if they had not been retracted, distorting the user's judgment.

Finally, postpublication peer review is an interesting means for strengthening scientific ethics, as it extends the critical review process beyond the initial journal screening and enables the ongoing correction of errors, biases, or potential fraud. Unlike traditional peer review, which is limited in terms of both the number of experts involved and the time available, this model opens the discussion to a broader community, thereby promoting transparency and reproducibility while facilitating the rapid detection of inconsistencies. Studies have shown that this type of review improves the quality of scientific debate and helps to identify issues that were overlooked during the initial review [51].

## 5 Conclusion: Maintaining Scientific Publication as the Benchmark for Scientific Evidence

Scientific publication is the central pillar for the validation and dissemination of scientific knowledge, as the acceleration of discoveries and new modes of communication via social networks require solid benchmarks to distinguish opinion from proven fact. Despite its limitations, the peer‐review model guarantees methodological quality and a solid theoretical corpus, ensuring that reported results will be reproducible. Faced with the rise of preprints and scientific social networks, maintaining a rigorous editorial framework is crucial to avoid dilution of the scientific signal in the informational noise. Some private research organizations are tempted to communicate their research findings outside of any academic framework. For example, Elon Musk's Neuralink company implanted a “brain‐reading” device into a person for the first time, according to brief communications on social media. This brain‐computer interface experiment was carried out without registering the study at ClinicalTrials.gov, the online repository curated by the US National Institutes of Health. Moreover, the first human trials were announced without any scientific publication evaluated by independent scientists. This deliberate circumvention of scientific publication to present unverified or “alternative” scientific data is worrying.

The US Academies of Science, Engineering, and Medicine have undertaken a review to reach a consensus on correcting scientific data [52]. The committee will examine practices in different countries, with recommendations to improve and make them transparent, which can, ideally, be applied by all those involved in scientific publishing. These recommendations will particularly encourage all scientific journals to implement rigorous reproducibility policies. This will include specific editorial guidelines and requirements for sharing study protocols and data, as well as the preregistration of studies. It is to be hoped that this US committee will be able to maintain its scientific and intellectual autonomy. Publication is undoubtedly the gold standard for the dissemination of scientific evidence, but it needs to evolve to survive.

## Author Contributions

**Luc Zimmer:** conceptualization, writing – original draft, writing – review and editing, supervision. **Pascal Bousquet:** writing – original draft, writing – review and editing.

## Funding

The authors have nothing to report.

## Ethics Statement

The authors have nothing to report.

## Conflicts of Interest

The authors declare no conflicts of interest.

## Data Availability

This article does not report new data. All data discussed in the paper are publicly available in the originally referenced publications.
